# Nationwide seroprevalence of SARS-CoV-2 and identification of risk factors in the general population of the Netherlands during the first epidemic wave

**DOI:** 10.1136/jech-2020-215678

**Published:** 2020-11-30

**Authors:** Eric R A Vos, Gerco den Hartog, Rutger M Schepp, Patricia Kaaijk, Jeffrey van Vliet, Kina Helm, Gaby Smits, Alienke Wijmenga-Monsuur, Janneke D M Verberk, Michiel van Boven, Rob S van Binnendijk, Hester E de Melker, Liesbeth Mollema, Fiona R M van der Klis

**Affiliations:** Centre for Infectious Disease Control, RIVM, Bilthoven, Netherlands

**Keywords:** Epidemics, Epidemiology, Infection, Public health, Surveillance

## Abstract

**Background:**

We aimed to detect SARS-CoV-2 serum antibodies in the general population of the Netherlands and identify risk factors for seropositivity amidst the first COVID-19 epidemic wave.

**Methods:**

Participants (n=3207, aged 2–90 years), enrolled from a previously established nationwide serosurveillance study, provided a self-collected fingerstick blood sample and completed a questionnaire (median inclusion date 3 April 2020). IgG antibodies targeted against the spike S1-protein of SARS-CoV-2 were quantified using a validated multiplex-immunoassay. Seroprevalence was estimated controlling for survey design, individual pre-pandemic concentration, and test performance. Random-effects logistic regression identified risk factors for seropositivity.

**Results:**

Overall seroprevalence in the Netherlands was 2.8% (95% CI 2.1 to 3.7), with no differences between sexes or ethnic background, and regionally ranging between 1.3 and 4.0%. Estimates were highest among 18–39 year-olds (4.9%), and lowest in children 2–17 years (1.7%). Multivariable analysis revealed that persons taking immunosuppressants and those from the Orthodox-Reformed Protestant community had over four times higher odds of being seropositive compared to others. Anosmia/ageusia was the most discriminative symptom between seropositive (53%) and seronegative persons (4%, p<0.0001). Antibody concentrations in seropositive persons were significantly higher in those with fever or dyspnoea in contrast to those without (p=0.01 and p=0.04, respectively).

**Conclusions:**

In the midst of the first epidemic wave, 2.8% of the Dutch population was estimated to be infected with SARS-CoV-2, that is, 30 times higher than reported. This study identified independent groups with increased odds for seropositivity that may require specific surveillance measures to guide future protective interventions internationally, including vaccination once available.

## INTRODUCTION

Severe acute respiratory syndrome coronavirus 2 (SARS-CoV-2), causative agent of coronavirus disease (COVID-19), emerged in Wuhan, China, in late 2019. On 11 March 2020, the World Health Organization (WHO) declared COVID-19 a pandemic, with over 10 million confirmed cases as of the beginning of July 2020.^1 2^ The first patient in the Netherlands was confirmed on 27 February 2020.^3^ Cases primarily clustered in the southeastern part of the country, but were reported in other regions quickly hereafter. Multi-pronged interventions to suppress the spread of the virus, including social distancing, school and bar/restaurant closure, and stringent advice to home quarantine when feeling ill and work from home, were implemented on 16 March 2020—and were relaxed gradually since 1 June 2020. By 1 July 2020, 50 273 cases, 11 877 hospitalisations, and 6113 related deaths were reported in the Netherlands.^3^

10.1136/jech-2020-215678.supp2 Supplementary data

Reported COVID-19 cases worldwide are an underestimation of the true magnitude of the pandemic. The scope of undetected cases remains largely unknown due to difference in restrictive testing policy and registration across countries, and occurrence of asymptomatic infections.^4 5^ Large-scale nationwide serosurveillance studies measuring SARS-CoV-2-specific serum antibodies could help to better assess the number of infections, viral spread, and groups at risk of infection in the general population by incorporating extensive questionnaire data, for example, on lifestyle, behaviour and profession. This might yield different factors than those identified for (severely-ill) clinical cases investigated more frequently up until now.^6 7^ Unfortunately, such nationwide studies (eg, in Spain^8^ and Iceland,^9^) also referred to as Unity Studies by the WHO,^10^ are scarce and mainly set up through convenience sampling.

Therefore, a nationwide serosurveillance study (PIENTER-Corona, PICO) was initiated quickly after the lockdown was in effect. This cohort is unique as it comprises data available from a previous serosurvey established in 2016/17 (PIENTER-3) of a randomised nationwide sample of Dutch citizens, across all ages and a separate sample enriched for Orthodox-Reformed Protestants, whom might have been exposed to SARS-CoV-2 more frequently due to their socio-geographical-clustered lifestyle.^11 12^ The presented serological framework and findings of our first round of inclusion can support public health policy in the Netherlands as well as internationally.

## METHODS

### Study design

**Figure 1:**
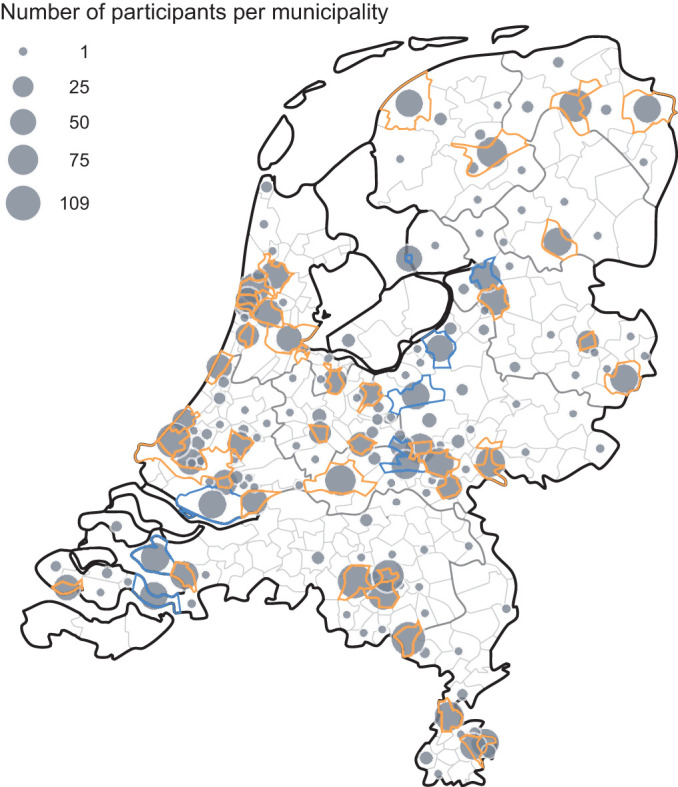
Geographical representation of number of participants in the PICO-study, the Netherlands, first round of inclusion, per municipality. The size of the dots reflect the absolute number of participants. Thicker grey and smaller light grey boundaries represent provinces and municipalities, respectively, and orange and blue boundaries characterise municipalities from the national and low vaccination coverage sample, respectively.

In 2016/17, the National Institute for Public Health and the Environment of the Netherlands (RIVM) initiated a large-scale nationwide serosurveillance study (PIENTER-3) (n=7600; age-range 0–89 years). The primary aim was to obtain insights into the protection against vaccine-preventable diseases offered by the National Immunisation Programme in the Netherlands. A comprehensive description of PIENTER-3 has been published previously.^13^ Briefly, participants were selected via a two-stage cluster design, comprising 40 municipalities in five regions nationwide (henceforth ‘national sample’, NS), and nine municipalities in the low vaccination coverage municipalities (LVC), inhabited by a relative large proportion of Orthodox-Reformed Protestants (figure 1). Among other materials, sera and questionnaire data had been collected from all participants. Hence, the PIENTER-3 study acted as baseline sample of the Dutch population for the present cross-sectional PICO-study since 6102 participants (80%) consented to be approached for follow-up (after updating addresses and screening of possible deaths). The study was powered to estimate an overall seroprevalence with a precision of at least 2.5%.^13^ The PICO-study protocol was approved by the Medical Ethics Committee MEC-U, the Netherlands (Clinical Trial Registration NTR8473), and conformed to the principles embodied in the Declaration of Helsinki.

### Study population and materials

On 25 March 2020, an invitation letter was sent. Invitees (age-range 2–92 years) willing to participate registered online. After enrolment, participants received an instruction letter on how to self-collect a fingerstick blood sample in a microtainer (maximum of 0.3 mL). Blood samples were returned to the RIVM-laboratory in safety envelopes. Serum samples were stored at −20°C awaiting analyses. Materials were collected between March 31 and May 11, with the majority (80%) in the first week of April 2020 (median collection date April 3). Simultaneous with the blood collection, participants were asked to complete an (online) questionnaire, including questions regarding sociodemographic characteristics, COVID-19-related symptoms, and potential other determinants for SARS-CoV-2 seropositivity, such as comorbidities, medication use and behavioural factors. All participants provided written informed consent.

### Laboratory methods

Serum samples (diluted 1:200) were tested for the presence of SARS-CoV-2 spike S1-specific IgG antibodies using a validated fluorescent bead-based multiplex-immunoassay as described.^14^ A cut-off concentration for seropositivity (2.37 AU/mL; with specificity of 99% and sensitivity of 84.4%) was determined by ROC-analysis of 400 pre-pandemic control samples (including a nationwide random cross-sectional sample (n=108)) as well as patients with confirmed influenza-like illnesses caused by coronaviruses and other viruses, and a selection of sera from 115 PCR-confirmed COVID-19 cases with mild, or severe disease symptoms. Seropositive PICO-samples and those with a concentration 25% below the cut-off were retested (n=138), and the geometric mean concentration (GMC) was calculated. Paired pre-pandemic PIENTER-3-samples of these retested PICO-samples (available from 129/138) were tested correspondingly as described above to correct for false-positive results (online supplemental figure S1A).

### Statistical analyses

#### Study population, COVID-19-related symptoms and antibody responses

Data management and analyses were conducted in SAS v.9.4 (SAS Institute Inc., USA) and R v.3.6. P values <0.05 were considered statistically significant. Sociodemographic characteristics and COVID-19-related symptoms (general, respiratory, and gastrointestinal) developed since the start of the epidemic were stratified by sample (NS vs LVC), or sex, respectively, and described for seropositive and seronegative participants. Differences were tested via Pearson’s χ², or Fisher’s exact test if appropriate. Differences in GMC between reported symptoms in seropositive participants were determined by calculating the difference in log-transformed concentrations of those who developed symptoms at least 4 weeks prior to the sampling—ensuring a plateaued response—and tested by means of a Mann-Whitney U-test.

#### Seroprevalence estimates

Seroprevalence estimates (with 95% Wilson CIs (CI)) for SARS-CoV-2-specific antibodies were calculated taking into account the survey design (ie, controlling for region and municipality) and weighted by sex, age, ethnic background and degree of urbanisation to match the distribution of the general Dutch population in both the NS and LVC sample. Estimates were corrected for test performance via the Rogan & Gladen bias correction (with sensitivity of 84.4% and assuming a specificity of 100% after cross-validation with pre-sera).^15^ Smooth age-specific seroprevalence estimates were obtained with a logistic regression in a Generalised Additive Model using penalised splines.^16^

#### Risk factors for SARS-CoV-2 seropositivity

A random-effects logistic regression model was used to identify risk factors for SARS-CoV-2 seropositivity, applying a full case analysis (n=3100; values were missing for <5% of the participants). Potential risk factors included sociodemographic characteristics (sex, age group, region, ethnic background, Orthodox-Reformed Protestants, educational level, household size, (parent with a) contact profession, healthcare worker), and COVID-19-related factors (contact with a COVID-19 confirmed case, number of persons contacted yesterday, working from home (normally and in the last week), comorbidities (combining diabetes, history of malignancy, immunodeficiency, cardio-vascular, kidney and chronic lung disease (note: as a sensitivity analysis, comorbidities were also included separately)), and use of blood pressure medication, immunosuppressants, statins and antivirals/antibiotics in the last month). Models included a random intercept, potential clustering by municipality and region was accounted for, and odds ratios (OR) in univariable analyses were a priori adjusted for sex and age. Variables with p<0.10 were entered in the multivariable analysis, and backward selection was performed—manually dropping variables one-by-one based on p≥0.05—to identify significant risk factors. Adjusted ORs and corresponding 95% CIs were provided.

## RESULTS

### Study population

Of 6102 invitees, 3207 (53%) donated a serum sample and filled-out the questionnaire, of which 2637 persons from the NS and 570 from the LVC. Participants from across the country participated (figure 1), with age ranging from 2 to 90 years (table 1). In the NS, slightly more women (55%) participated, most (88%) were of Dutch descent, nearly half had a high educational level, and 45% was religious. 20 percent of persons between age 25–66 years were healthcare workers and 56% of the (parents of) participants reported to have had daily contact with patients, clients and/or children in their profession/volunteer work normally. Over half of the participants lived in a ≥2-person household, and 78% reported to have had physical contact with <5 people outside their own household yesterday (during lockdown), of which more than half with nobody. Comorbidities most frequently reported included chronic lung and cardiovascular disease (both 13%), and a history of malignancy (5%). In line with the population distribution, the LVC sample was characterised by a relative high proportion of Orthodox-Reformed Protestants from Dutch descent (table 1). Sociodemographic characteristics between responders and non-responders are provided in online supplemental table S1.

**Table 1:** Sociodemographic characteristics of participants in the PICO-study and weighted seroprevalence in the general population of the Netherlands, first round of inclusion, by national sample and low vaccination coverage sample

			National sample				Low vaccination coverage sample			
			Total		Weighted SARS-CoV-2 seroprevalence		Total		Weighted SARS-CoV-2 seroprevalence	
			n	%	%	95% CI	n	%	%	95% CI
**Overall**			2637	100	2.8	2.1–3.7	570	100	2.9	1.4–6.3
**Sex**										
	Men		1184	44.9	2.9	1.8–4.5	233	40.9	4.0	1.5–10.6
	Women		1453	55.1	2.7	1.7–4.1	337	59.1	1.9	0.7–4.9
**Age categories**										
	2–17		507	19.2	1.7	0.6–4.9	93	16.3	0.0	NA
	18–39		735	27.9	4.9	3.2–7.5	196	34.4	6.8	3.0–14.6
	40–64		919	34.8	1.9	1.2–3.2	198	34.7	2.4	0.7–8.3
	65–90		476	18.1	2.5	1.2–5.1	83	14.6	1.0	0.1–7.0
**Region**										
	North		566	21.5	1.3	0.4–3.2	NA	NA	NA	NA
	Mid-West		427	16.2	4.0	1.8–8.0	NA	NA	NA	NA
	Mid-East		508	19.3	3.1	1.3–6.2	NA	NA	NA	NA
	South-West		468	17.7	3.0	1.5–5.3	NA	NA	NA	NA
	South-East		668	25.3	2.7	1.4–4.7	NA	NA	NA	NA
	Low vaccination coverage municipalities		NA	NA	NA	NA	570	100	2.9	1.4–6.3
**Ethnicity**										
	Dutch		2306	87.5	2.8	2.0–3.7	555	97.4	3.0	1.4–6.5
	Non-Dutch Western		159	6.0	2.0	0.6–7.1	12	2.1	0.0	NA
	Non-Western		172	6.5	3.4	1.4–8.4	3	0.5	0.0	NA
Educational level*										
	High		1257	46.7	2.5	1.6–3.9	173	30.7	2.3	0.5–9.4
	Middle		883	34.2	3.5	2.0–6.2	252	44.8	4.4	1.7–10.9
	Low		442	17.1	2.2	1.0–5.0	138	24.5	0.9	0.1–6.3
**Religion**										
	No religion		1329	54.5	2.9	1.9–4.4	145	28.0	0.3	0.0–3.7
	Roman Catholic		613	25.1	3.4	1.7–6.6	13	2.5	0.0	NA
	Other		119	4.9	0.0	NA	14	2.7	0.0	NA
	Protestant		379	15.5	3.0	1.6–6.4	346	66.8	3.7	1.5–8.8
		Orthodox-Reformed	28	7.4	8.5	2.4–26.9	102	29.5	7.4	1.8–26.8
		Other	351	92.6	2.6	1.0–6.5	244	70.5	2.2	0.9–5.3

NA, Not applicable.

*Maternal educational level was used for participants <15 years of age.

Missing: in the national sample: (maternal) educational level=55, religion=197; in the low vaccination coverage sample: (maternal) educational level=7, religion: 52.

10.1136/jech-2020-215678.supp1 Supplementary data

### COVID-19-related symptoms and antibody responses

In total, 63% of participants reported to have had ≥1 COVID-19-related symptom(s) since the start of the epidemic, with runny nose (37%), headache (33%), and cough (30%) being most common (table 2). All reported symptoms were significantly higher in seropositive compared to seronegative persons, except for stomach ache. The majority of those seropositive (93%) reported to have had symptoms (90% of men vs 95% of women), of whom three already in mid-February, 2 weeks prior to the official first notification. Median duration of illness in the seropositive participants was 8.5 days (IQR: 4.0–12.5), 16% (n=12) visited ageneral practitioner and one was admitted to the hospital. Among seropositive persons, most reported to have had ≥1 respiratory symptom(s) (86%), with runny nose and cough (both 61%) most regularly, and ≥1 general (84%) symptom(s), of which anosmia/ageusia (53%) was most discriminative as compared to the seronegative participants (4%, p<0.0001) (table 2). Symptoms were more common in women, except for anosmia/ageusia, cough and irritable/confusion. Almost 75% of the seropositive participants met the COVID-19 case definition of fever and/or cough and/or dyspnoea, which improved to 80% when anosmia/ageusia was included—while remaining 36% in those seronegative. GMC was significantly higher among seropositive persons with fever vs without (48.2 vs 11.6 AU/mL, p=0.01), and with dyspnoea vs without (78.6 vs 13.5 AU/mL, p=0.04).

**Table 2:** COVID-19-related symptoms since the start of the epidemic among all participants in the PICO-study reporting symptoms (n=3147), first round of inclusion

		SARS-CoV-2 seronegative n=3073		SARS-CoV-2 seropositive n=74		Total n=3147		
		n	%	n	%	n	%	P value*
**Meets COVID-19 case definition**								<0.0001
	Yes	1096	35.7	55	74.3	1151	36.6	
	No	1977	64.3	14	25.7	1996	63.4	
**Meets COVID-19 case definition, and self-reported to have had anosmia and/or ageusia**								<0.0001
	Yes	1113	36.2	59	79.7	1172	37.2	
	No	1960	63.8	15	20.3	1975	62.8	
**Developed symptoms since the start of the epidemic**								<0.0001
	Yes	1903	61.9	69	93.2	1972	62.7	
	No	1170	38.1	5	6.8	1175	37.3	
**General symptoms (one or more)**		1350	43.9	62	83.8	1412	44.9	<0.0001
	Fever	361	11.8	32	43.2	393	12.5	<0.0001
	General malaise	332	10.8	34	46.0	366	11.6	<0.0001
	Headache	1001	32.6	48	64.9	1049	33.3	<0.0001
	Irritable/confused	232	7.6	17	23.0	249	7.9	<0.0001
	Muscle ache	312	10.5	22	29.7	334	10.6	<0.0001
	Arthralgia	497	16.2	42	56.8	539	17.1	<0.0001
	Anosmia and/or ageusia	111	3.6	39	52.7	150	4.8	<0.0001
**Respiratory symptoms (one or more)**		1622	52.8	64	86.5	1686	53.6	<0.0001
	Cough	905	29.5	45	60.8	950	30.2	<0.0001
	Sore throat	798	26.0	33	44.6	831	26.4	0.0003
	Runny nose	1128	36.7	45	60.8	1173	37.3	<0.0001
	Solely a runny nose & hay fever	22	0.7	1	1.4	23	0.7	0.42†
	Dyspnoea	251	8.2	13	17.6	264	8.4	0.004
**Gastrointestinal symptoms (one or more)**		668	21.7	32	43.2	700	22.2	<0.0001
	Diarrhoea	388	12.6	18	24.3	406	12.9	0.003
	Nausea/vomiting	207	6.7	13	17.6	220	7.0	0.0003
	Stomach ache	364	11.9	13	17.6	377	12.0	0.13

*p values were calculated with Pearson’s χ² Test, unless depicted otherwise.

†p value was calculated with Fisher’s Exact Test.

Missing values for all symptoms: 60.

### Seroprevalence estimates

**Figure 2 F2:**
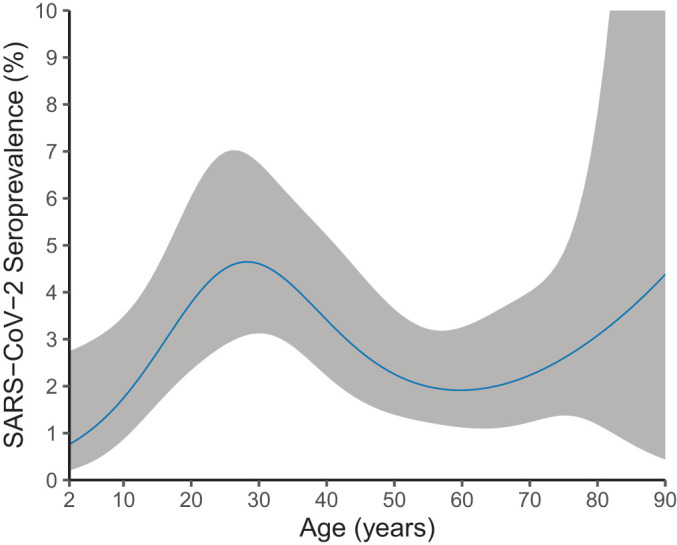
Smooth age-specific SARS-CoV-2 seroprevalence in the general population of the Netherlands, beginning of April 2020.

Overall weighted seroprevalence in the NS was 2.8% (95% CI 2.1 to 3.7), did not differ between sexes or ethnic backgrounds (table 1), and was not higher among healthcare workers (2.7% vs non-healthcare workers 2.5%). Seroprevalence was lowest in the northern region (1.3%) and highest in the mid-west (4.0%). Estimates were lowest in children—gradually increasing from below 1% at age 2 years to 3% at 17 years—was highest in age group 18–39 years (4.9%) and ranged between 2 and 4% up to 90 years of age (figure 2). In both samples, seroprevalence was highest in Orthodox-Reformed Protestants (>7%) (table 1). Online supplement figure S1B displays the distribution of IgG concentrations for all participants by age, and online supplemental figure S2 [Fig F2]shows the seroprevalence smoothed by age in the LVC.

### Risk factors for SARS-CoV-2 seropositivity

Variables that were associated with SARS-CoV-2 seropositivity in univariable analyses included age group, Orthodox-Reformed Protestant, had been in contact with a COVID-19 case, use of immunosuppressants, and antibiotic/antiviral medication in the last month (table 3). In multivariable analysis, substantial higher odds were observed for those who took immunosuppressants the last month, were Orthodox-Reformed Protestant, had been in contact with a COVID-19 confirmed case, and from age groups 18–24 and 25–39 years (compared to 2–12 years).

**Table 3:** Risk factor analysis for SARS-CoV-2 seropositivity among all participants (n=3100; full case analysis) in the PICO-study, first round of inclusion

			% SARS-CoV-2 seropositive		Univariable model*				Multivariable model			
		n_total_	n	%	OR	95% CI		P value	Adjusted OR	95% CI		P value
**Age group**								0.016				0.105
	2–12	457	4	0.9	Ref.				Ref.			
	13–17	129	1	0.8	0.88	0.10	7.91		0.87	0.10	7.91	
	18–24	226	12	5.3	6.47	2.05	20.43		4.52	1.40	14.58	
	25–39	696	24	3.5	4.17	1.43	12.14		3.10	1.05	9.14	
	40–49	429	11	2.6	3.05	0.96	9.68		2.48	0.77	7.98	
	50–59	485	8	1.7	1.94	0.58	6.49		1.49	0.44	5.07	
	60–69	377	7	1.9	2.16	0.63	7.44		1.71	0.49	5.98	
	70–90	301	7	2.3	2.64	0.76	9.14		2.46	0.70	8.60	
**Sex**								0.81				0.57
	Men	1368	32	2.3	Ref.				Ref.			
	Women	1732	42	2.4	1.06	0.66	1.71		1.15	0.71	1.88	
**Region**†								0.64				0.36
	North	537	7	1.3	Ref.				Ref.			
	Mid-West	411	11	2.7	2.14	0.80	5.72		2.27	0.86	5.98	
	Mid-East	494	14	2.8	2.27	0.89	5.80		2.00	0.79	5.04	
	South-West	451	11	2.4	1.83	0.69	4.86		1.80	0.69	4.74	
	South-East	652	17	2.6	2.04	0.82	5.07		2.08	0.85	5.12	
	LVC	555	14	2.5	1.80	0.71	4.61		1.09	0.40	2.94	
**Orthodox-Reformed Protestant**								0.001				0.0007
	No	2972	65	2.2	Ref.				Ref.			
	Yes	128	9	7.0	4.04	1.72	9.48		4.50	1.89	10.74	
**Had been in contact with a COVID-19 confirmed case**								<0.0001				<0.0001
	No	2074	33	1.6	Ref.				Ref.			
	Yes	192	16	8.3	4.65	2.44	8.87		4.97	2.58	9.56	
	Don’t know	834	25	3.0	1.75	1.03	2.99		1.88	1.10	3.22	
**Took immunosuppressants, last month**								0.006				0.001
	No	3039	69	2.3	Ref.				Ref.			
	Yes	61	5	8.2	3.94	1.50	10.39		5.05	1.89	13.48	
**Took antibiotics/antiviral medication, last month**								0.01				
	No	2901	64	2.2	Ref.							
	Yes	199	10	5.0	2.43	1.21	4.89					

*Variables that were not associated with SARS-CoV-2 seropositivity in univariable analyses (ie, p≥0.10)—or that were not controlled for—included: ethnic background, (maternal) educational level, household size, (parent with a) contact profession, healthcare worker, number of persons contacted yesterday, working from home (normally and in the last week (during lockdown)), comorbidities (combining chronic lung disease, diabetes, history of malignancy, immunodeficiency, cardio-vascular disease, kidney disease), and use of blood pressure medication, immunosuppressants, statins and antivirals/antibiotics in the last month.

†Region North comprised provinces Groningen, Friesland, Drenthe and Overijssel, region Mid-West provinces Noord-Holland and Flevoland, region Mid-West provinces Utrecht and Gelderland, region South-West provinces Zuid-Holland and Zeeland, and region South-East provinces Noord-Brabant and Limburg.

CI, Confidence interval; LVC, Low vaccination coverage municipalities; OR, Odd ratio; Ref., Reference category.

## DISCUSSION

Here, we have estimated the seroprevalence of SARS-CoV-2-specific antibodies and identified risk factors for seropositivity in the general population of the Netherlands during the first epidemic wave in April 2020. Although overall seroprevalence was still low at this phase, important risk factors for seropositivity could be identified, including adults aged 18–39 years, persons using immunosuppressants, and Orthodox-Reformed Protestants. These data can guide future interventions, including strategies for vaccination, believed to be a realistic solution to overcome this pandemic.

This PICO-study revealed that 2.8% (95% CI 2.1 to 3.7) of the Dutch population had detectable SARS-CoV-2-specific serum IgG antibodies, suggesting that almost half a million inhabitants (of in total 17 423 981^17^) were infected (487 871 (95% CI 365 904 to 644 687)) in mid-March, 2020 (taking into account the median time to seroconvert^18^). Several seropositive participants reported to have had COVID-19-related symptoms back in mid-February, suggesting the virus circulated in our country at the beginning of February already. Our overall estimate is in line with preliminary results from another study conducted in the Netherlands in the beginning of April which found 2.7% to be seropositive, although this study was performed in healthy blood donors aged 18–79 years.^19^ Worldwide, various seroprevalence studies are ongoing. A large nationwide study in Spain showed that around 5% (ranging between 3.7% and 6.2%) was seropositive, indicating that only a small proportion of the population had been infected in one of the hardest hit countries in Europe. Current studies in literature mostly cover COVID-19 hotspots or specific regions—with possibly bias in selection of participants and/or smaller age-ranges—with rates ranging between 1–7% in April (eg, in Los Angeles County (CA, USA)^20^ or ten other sites in the USA,^21^ Geneva (Switzerland),^22^ and Luxembourg^23^). Estimates also very much depend on test performances. Particularly, when seroprevalence is relatively low, specificity of the assay should approach near 100% to diminish false-positive results and minimise overestimation. Although we cannot rule-out false-positive samples completely, our assay was validated using a broad range of positive and negative SARS-CoV-2 samples; PICO-samples were cross-linked to pre-pandemic concentration; and bias correction for test performance was applied to represent most accurate estimates. In addition, future studies should establish whether epidemiologically dominant genetic changes in the spike protein of SARS-CoV-2 influence binding to spike S1 used in our and other assays.

Seroprevalence was highest in adults aged 18–39 years, which is in line with the serosurvey among blood donors in the Netherlands, but contrary to the low incidence rate as reported in Dutch surveillance, caused by restrictive testing of risk groups and healthcare workers at the beginning of the epidemic, primarily identifying severe cases.^3 19^ The elevation in these younger adults may be explained by increased social contacts typical for this age group, in addition to specific social activities in February, such as skiing holidays in the Alps (from where the virus disseminated quickly across Europe), or carnival festivities in the Netherlands (ie, multiple superspreading events primarily in the mid and Southern part, explaining local elevation in seroprevalence). In correspondence with other nationwide studies^8 9^ and reports from the Dutch government,^3 24^ seroprevalence was lowest in children. Although some rare events of paediatric inflammatory multisystem syndrome have been reported, this group seems to be at decreased risk for developing (severe) COVID-19 in general, which may be explained by less severe infection possibly resulting in a limited humoral response.^25 26^ Further, significantly higher odds for seropositivity were seen in Orthodox-Reformed Protestants. This community lives socio-geographically clustered in the Netherlands, that is, work, school, leisure and church are intertwined heavily. As observed in other countries, particularly frequent attendance of church with close distance to others, including singing activities, might have fuelled the spread of SARS-CoV-2 within this community in the beginning of the epidemic.^11 12^ Whereas the comorbidities with possible increased risk of severe COVID-19 were not associated with seropositivity in this study, immunosuppressants use did display higher odds (note: we did not have information of specific drugs). Recent data indicate that immunosuppressive treatment is not associated with worse COVID-19 outcomes,^27 28^ yet continued surveillance is warranted as these patients might be more prone to (future) infection, for instance due to a possible attenuated humoral immune response.^29^

The majority of seropositive participants exhibited ≥1 symptom(s), mostly general and respiratory. A recent meta-analysis found a pooled asymptomatic proportion of 16%,^5^ hence the observed overall fraction in the present study (7%) might be a conservative estimate as the self-reported symptoms could have been due to other reasons or circulating pathogens along the recalled period (ie, 62% of the seronegative participants reported symptoms too). The asymptomatic proportion might be different across ages^5^ and should be explored further along with elucidating the overall contribution of asymptomatic transmission via well-designed contact-tracing studies. Interestingly, clinical studies have observed anosmia/ageusia to be associated with SARS-CoV-2 infection, and this notion is supported here at a population-based level.^30^ In the pandemic context, sudden onset of anosmia/ageusia seems to be a useful surveillance tool, which can contribute to early disease recognition and minimise transmission by rapid self-isolation.

This study has some limitations. First, although half of the total municipalities in the Netherlands were included, some COVID-19 hotspots might be missed due to the study design. Second, our study population consisted of more Dutch (88%) than non-Dutch persons and relative more healthcare workers (20%) when compared to the general population (76% and 14%, respectively).^17^ Healthcare workers in the Netherlands do not seem to have had a higher likelihood of infection, and transmission seems to have taken place mostly in household settings.^3 31^ Although selectivity in response was minimised by weighting our study sample on a set of sociodemographic characters to match the Dutch population, seroprevalence might still be slightly influenced. Third, some potential determinants for seropositivity could have been missed as we might have been underpowered to detect small differences given the low prevalence in this phase, or because these questions had not been included in the questionnaire (as it was designed in the very beginning of the epidemic). Finally, at this stage the proportion of infected individuals that fail to show detectable seroconversion is unknown, potentially leading to underestimation of the percentage of infected persons.

To conclude, we estimated that 2.8% of the Dutch inhabitants, that is, nearly half a million, were infected with SARS-CoV-2 amidst the first epidemic wave in the beginning of April 2020. This is in striking contrast with the 30-fold lower number of reported cases (of approximately 15 000)^3^, and underlines the importance of seroepidemiological studies to estimate the true pandemic size. The proportion of persons still susceptible to SARS-CoV-2 is high and IFR is substantial.^4^ Globally, nationwide seroepidemiological studies are urgently needed for better understanding of related risk factors, viral spread, and measures applied to mitigate dissemination.^7^ The prospective nature of our study will enable us to gain key insights on the duration and quality of antibody responses in infected persons, and hence possible protection of disease by antibodies.^6^ Serosurveys will thus play a major role in guiding future interventions, such as strategies for vaccination (of risk groups), since even when vaccines become available, initial vaccine availability will be limited.

**What is already known on this topic**

- Reported COVID-19 cases worldwide are an underestimation of the true magnitude of the pandemic as the scope of undetected cases remains largely unknown.
- Various symptoms and risk factors have been identified in patients seeking medical advice, however, these may not be representative for infections in the general population.
- Seroepidemiological studies in outbreak settings have been performed, however, studies on a nationwide level covering all ages remain limited.

**What this study adds**

- This nationwide seroepidemiological study covering all ages reveals that 2.8% of the Dutch population had been infected with SARS-CoV-2 at the beginning of April 2020, that is, 30 times higher than the official cases reported, leaving a large proportion of the population still susceptible for infection.
- The highest seroprevalence was observed in young adults from 18 to 39 years of age and lowest in children aged 2 to 17 years, indicating marginal SARS-CoV-2 infections among children in general.
- Persons taking immunosuppressants as well as those from the Orthodox-Reformed Protestant community had over four times higher odds of being seropositive compared to others.
- The extend of the spread of SARS-CoV-2 and the risk groups identified here, can inform monitoring strategies and guide future interventions internationally.
