# Supplementary material for: Nationwide seroprevalence of SARS-CoV-2 and identification of risk factors in the general population of the Netherlands during the first epidemic wave
Source: J Epidemiol Community Health. 2020 Nov 30;75(6):489–95. doi: 10.1136/jech-2020-215678 (PMC8142429; doi:10.1136/jech-2020-215678)
Supplement: Supplementary data [file jech-2020-215678supp002.pdf]

**a**

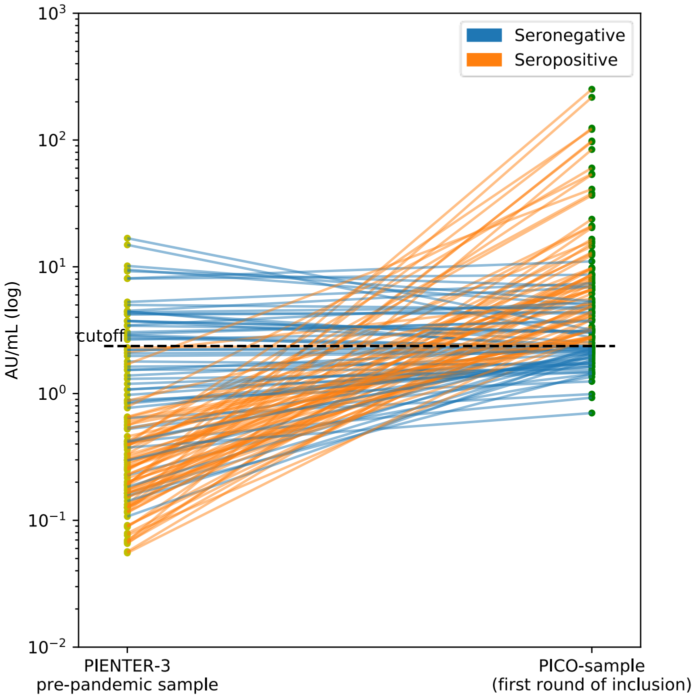


**b**

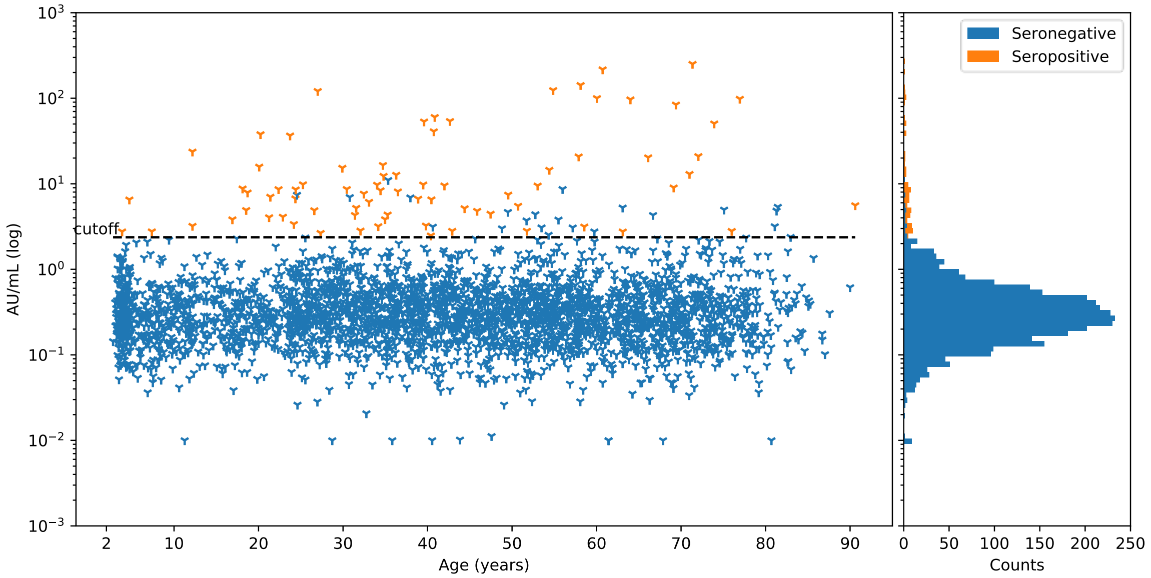


**FigureS1.** IgG antibody concentration (AU/mL) against SARS-CoV-2 of matched pre-pandemic PIENTER-3-samples (light green dots) and current PICO-samples (dark green dots) that were seropositive or 25% below the cutoff for seropositivity after the first measure (n=138) (**a**). PICO-samples with seropositive pre-pandemic sera (based on the calculated cutoff by ROC-analysis) were considered seronegative (n=26), thereby correcting for false-positivity. To note: a maximum concentration-fold increase of 1·5 was observed between a false-positive PIENTER-3-sample and its corresponding PICO-sample. Blue and orange lines represent seronegative and seropositive samples, respectively, and the dashed line depicted is considered the cutoff for seropositivity (2.37 AU/mL). (**b**) IgG antibody concentration (AU/mL (log)) against SARS-CoV-2 of all individual PICO-samples, by age (years) (left side) and distributed by means of a histogram (right side). After correction for pre-pandemic cross-reactivity, samples were classified as seronegative (blue) and seropositive (orange). The dashed line depicted is considered the cutoff for seropositivity (2.37 AU/mL).

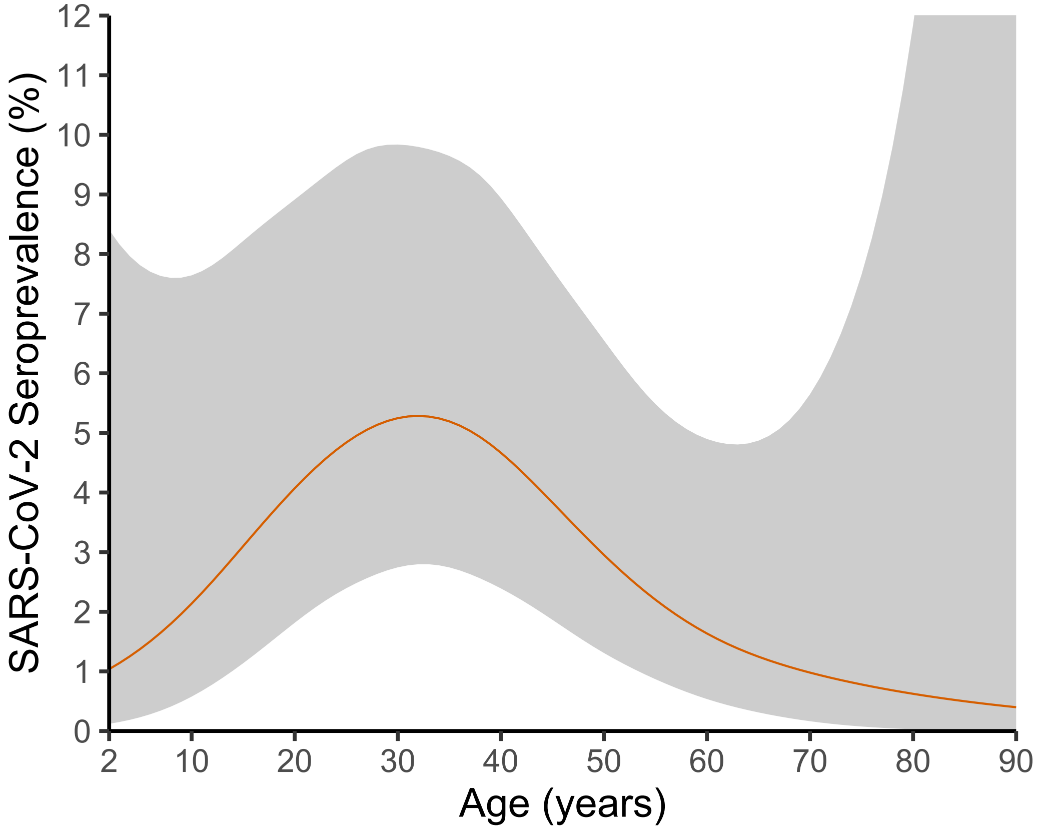


**FigureS2**. Smooth age-specific SARS-CoV-2 seroprevalence in the low vaccination coverage municipalities of the Netherlands, beginning of April 2020.
