# Supplementary material for: Nationwide seroprevalence of SARS-CoV-2 and identification of risk factors in the general population of the Netherlands during the first epidemic wave
Source: J Epidemiol Community Health. 2020 Nov 30;75(6):489–95. doi: 10.1136/jech-2020-215678 (PMC8142429; doi:10.1136/jech-2020-215678)
Supplement: Supplementary data [file jech-2020-215678supp001.pdf]

Supplement TableS1. Sociodemographic characteristics of responders and non-responders in the PICO-study, first round of inclusion

	Non-responder		Responder		Total
	n	%	*n*	%	
**Sex**					
Men	1360	47.0	1417	44.2	2777
Women	1535	53.0	1790	55.8	3325
**Age categories, years**					
2-12	691	23.9	468	14.6	1159
13-17	238	8.2	132	4.1	370
18-24	269	9.3	232	7.2	501
25-39	580	20.0	699	21.8	1279
40-49	292	10.1	440	13.7	732
50-59	248	8.6	492	15.4	740
60-69	224	7.7	401	12.5	625
70-90	353	12.2	343	10.7	696
**Region**					
North	447	15.4	566	17.7	1013
Mid-West	395	13.6	427	13.3	822
Mid-East	414	14.3	508	15.8	922
South-West	483	16.7	468	14.6	951
South-East	598	20.7	668	20.8	1266
Low vaccination coverage municipalities	558	19.3	570	17.8	1128
**Ethnicity**					
Dutch	2168	74.9	2861	89.2	5029
Non-Dutch Western	166	5.7	171	5.3	337
Non-Western	560	19.4	175	5.5	735
**Educational level**[a]					
High	787	29.2	1262	41.8	2049
Middle	984	36.4	1122	37.1	2106
Low	930	34.4	637	21.1	1567
**Religion**					
No religion	1070	40.6	1474	49.8	2544
Roman Catholic	501	19.0	626	21.2	1127
Other (Islamic, Jewish, Buddhism, Hinduism, other)	379	14.4	133	4.5	512
Protestant	686	26.0	725	24.5	1411
Orthodox-Reformed	182	26.5	130	17.9	312
Other	504	73.5	595	82.1	1099

Missing: ethnicity=1, educational level=380, religion=508.

[a] Educational level during inclusion of the PIENTER-3 study (2016/17) was used for accurate comparison between responders and non-responders. Note: maternal educational level was used for participants <15 years of age.
